# Synthesis of borate cross-linked rhamnogalacturonan II

**DOI:** 10.3389/fpls.2015.00223

**Published:** 2015-04-21

**Authors:** Hiroya Funakawa, Kyoko Miwa

**Affiliations:** ^1^Division of Biosphere Science, Graduate School of Environmental Science, Hokkaido University, Sapporo, Japan; ^2^Precursory Research for Embryonic Science and Technology, Japan Science and Technology Agency, Kawaguchi, Japan

**Keywords:** *Arabidopsis thaliana*, borate, glycosyltransferase, homogalacturonan, rhamnogalacturonan-II, pectin, primary cell wall, transporter

## Abstract

In the present review, we describe current knowledge about synthesis of borate crosslinked rhamnogalacturonan II (RG-II) and it physiological roles. RG-II is a portion of pectic polysaccharide with high complexity, present in primary cell wall. It is composed of homogalacturonan backbone and four distinct side chains (A–D). Borate forms ester bonds with the apiosyl residues of side chain A of two RG-II monomers to generate borate dimerized RG-II, contributing for the formation of networks of pectic polysaccharides. In plant cell walls, more than 90% of RG-II are dimerized by borate under boron (B) sufficient conditions. Borate crosslinking of RG-II in primary cell walls, to our knowledge, is the only experimentally proven molecular function of B, an essential trace-element. Although abundance of RG-II and B is quite small in cell wall polysaccharides, increasing evidence supports that RG-II and its borate crosslinking are critical for plant growth and development. Significant advancement was made recently on the location and the mechanisms of RG-II synthesis and borate cross-linking. Molecular genetic studies have successfully identified key enzymes for RG-II synthesis and regulators including B transporters required for efficient formation of RG-II crosslinking and consequent normal plant growth. The present article focuses recent advances on (i) RG-II polysaccharide synthesis, (ii) occurrence of borate crosslinking and (iii) B transport for borate supply to RG-II. Molecular mechanisms underlying formation of borate RG-II crosslinking and the physiological impacts are discussed.

## Introduction

Plant cell wall is composed of not only polysaccharides and proteins, but it also contains essential minerals including calcium (Ca) and boron (B). It has already been demonstrated that both elements are required for formation of networks of pectic polysaccharides in primary cell wall. Pectic networks are formed through crosslinking of pectic polysaccharide chains, and these crosslinkings are established in two different domains of pectin; homogalacturonan (HG) by Ca divalent cations and in rhamnogalacturonan II (RG-II) by borate.

B is one of essential micronutrient for plants and occurrence of B deficiency has been reported in both nature and agricultural practice (Shorrocks, 1997). B deficiency generally impairs cell elongation rather than cell division in growing tissues of plants. Typical B deficiency symptoms include inhibition of root cell elongation, leaf cell expansion and pollen tube elongation (Dell and Huang, 1997).

**FIGURE 1:**
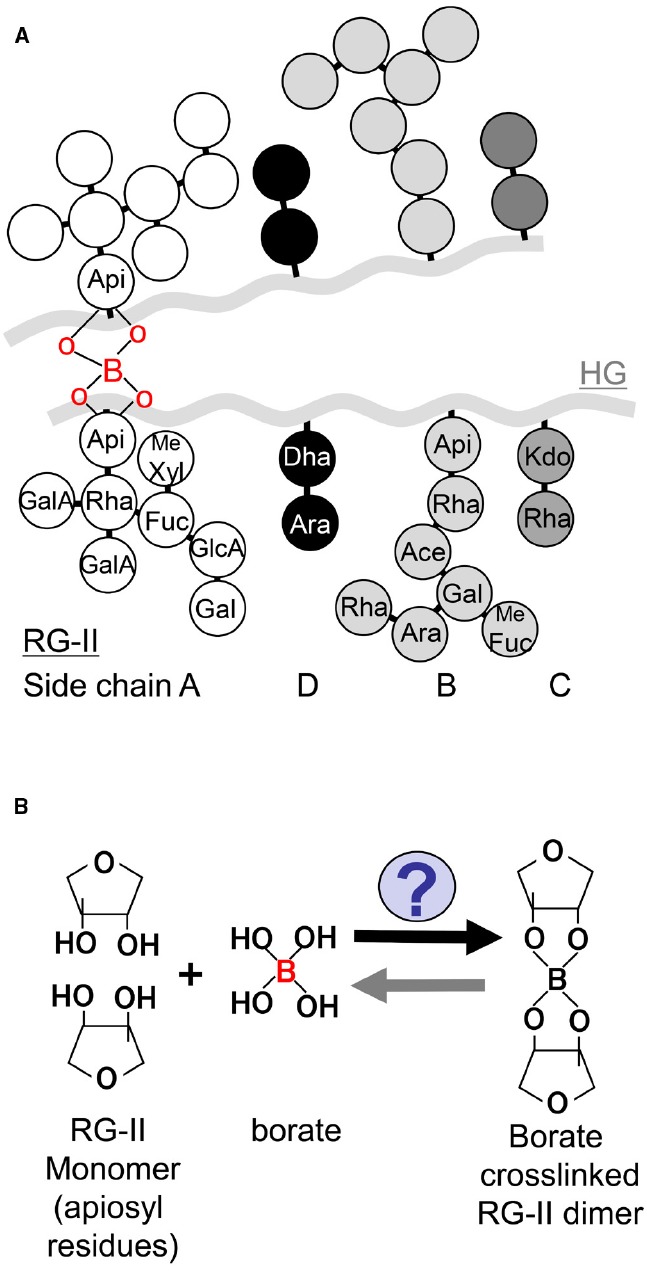
**Schematic model of RG-II structure and its borate crosslinking. (A)** A schematic model of RG-II structure. HG is a backbone of RG-II, and four distinct side chains (A–D) are linked to the backbone. Apiosyl residues in side chain A are covalently crosslinked by borate diester bond. Me Fuc: 2-*O*-methyl-L-fucose, Me Xyl: 2-*O*-methyl-D-xylose, Ace: aceric acid. (B) A schematic model of the reaction of borate dimerization of RG-II.

To our knowledge, borate crosslinking of RG-II in primary cell walls is only one proven function of B at the molecular level. Possible roles of B in membrane structure and in wall-membrane attachment have been also suggested (Blevins and Lukaszewski, 1998; Camacho-Cristóbal et al., 2008). The structure of RG-II-borate is well-characterized. RG-II is a pectic polysaccharide domain with high complexity, which is composed of HG backbone and four distinct side chains (A–D; Figure 1A). Borate forms ester bonds with the two apiosyl residues of side chain A of two RG-II monomers to generate borate dimerized RG-II, resulting in formation of networks of pectic polysaccharides (O’Neill et al., 2004). In plant cell walls, more than 90% of RG-II are dimerized by borate under B sufficient conditions. Abundance of RG-II is quite small in cell wall polysaccharides, however, increasing evidence supports that RG-II and its borate crosslinking are critical for plant growth and development by supporting cell adhesion and mechanical strength.

Then, how is such a complicated pectic polysaccharide RG-II synthesized and efficiently crosslinked by borate to be functional? The present article overviews recent advances on synthesis and physiological impacts of borate crosslinked RG-II in primary cell walls. Recent studies have provided novel clues to reveal molecular mechanisms of RG-II synthesis and subsequent borate crosslinking *in vitro* and *in vivo*. The article especially focuses on the processes in (i) RG-II polysaccharide synthesis, (ii) occurrence of borate crosslinking and (iii) B transport for borate supply to RG-II. Molecular genetic studies have uncovered glycosyl residues required for efficient borate dimerization in RG-II, through identification of enzymes involved in RG-II synthesis. Biochemical studies with the use of cultured cells suggest that borate RG-II dimerization occurs in the cytoplasm, possibly in Golgi or secretion pathways but not in apoplast, and also raise a new possibility that glycosylinositol phosphorylceramides could form a complex with RG-II to enhance borate dimerization of RG-II. Furthermore, studies on B transport also gave a new insight into preferential B distribution to cell walls and efficient dimerization of RG-II under limited B conditions for normal growth and development.

## Synthesis of RG-II Polysaccharides

Pectin is composed of three major polysaccharides; (i) HG, (ii) rhamnogalacturonan I (RG-I), and (iii) RG-II (Atmodjo et al., 2013). In brief, pectic polysaccharides are synthesized through two steps, (1) synthesis of nucleotide monosaccharides as activated-sugar donor substrates in cytoplasm and (2) transfer of activated-sugar to poly- or monosaccharides acceptor in Golgi apparatus. The synthesized polysaccharides are subsequently incorporated into cell walls.

A fundamental mystery for RG-II domain synthesis is the order of polysaccharide synthesis and organization of the four side chains. One possible hypothesis could be that synthesized side chains or monosaccharides of side chains are transferred to preexisting HG backbone. Another possibility could be galacturonic acids (GalA) present at the end of each side chain are linked after full-length side chains are individually synthesized. It might also be possible that a RG-II specific sugar works as a reaction origin and monosaccharides are sequentially transferred. To date, overall picture of RG-II synthesis remains unclear. Recent studies in *Arabidopsis thaliana* successfully led to identification of several enzymes involved in synthesis of HG, a backbone of RG-II, and RG-II side chains.

### Synthesis of HG by GAUTs

HG, a major component of pectin, is a polymer of (1 → 4)-α-linked D-galacturonic acids (D-GalA) and is also a backbone of RG-II. HG is synthesized in Golgi apparatus by HG;galacturonosyltransferase (HG;GalAT) which catalyzes elongation of HG by transferring D-GalA from UDP-D-GalA to oligogalacturonan (Sterling et al., 2001; Akita et al., 2002; Ishii, 2002). HG polysaccharides are secreted to apoplast and incorporated into cell walls after methyl-esterification at *O*-6 position. In cell wall, some regions of methyl-esterified HG is de-esterified or acetylated at *O*-2 or 3 position. The de-esterified HGs are cross-linked by Ca^2+^ to form gel and this cross-linkage is essential for mechanical strength and plant growth (Hongo et al., 2012).

Over the last decade, molecular identity of HG;GalAT has been revealed. In *A. thaliana*, GAlactUronosylTransferase (GAUT) 1 belonging to glycosyltransferase (GT) 8 family was first shown to exhibit HG;GalAT activity (Sterling et al., 2006). GAUT1 is localized in the Golgi lumen as a complex with anchor protein GAUT7 and catalyzes HG biosynthesis (Atmodjo et al., 2011). In addition, homology search based on amino acid sequence of the protein has revealed that 15 GAUT and 10 GAUT-like (GATL) families are present in *A. thaliana*, respectively (Sterling et al., 2006). Fifteen *AtGAUT* genes excluding *AtGAUT2* are ubiquitously expressed, and all 10 *AtGATL* genes are expressed with different tissues specificities (Caffall et al., 2009; Kong et al., 2011).

Until now, characterization of several mutants of *GAUT* and *GATL* genes has been conducted to depict their essential roles in cell morphology and physiological processes in *A. thaliana*. While either *gaut13* or *gaut14* single mutants did not show any morphological defects at whole plant level, inhibition of pollen tube elongation was found in the *gaut13 gaut14* double mutants (Wang et al., 2013). In immunohistochemical analysis, HG amount was observed to be reduced in the cell wall of pollen tube in the double mutant. These results suggest that *GAUT13* and *GAUT14* redundantly function in HG synthesis of pollen tube walls and pollen tube elongation. Considering that the cell wall of pollen tube is relatively rich in pectin, defects of HG synthesis are considered to be more evident in these cells.

Increasing number of reports have suggested that *GAUT* genes are involved in not only HG synthesis but also in synthesis of other cell wall components. Single mutants of *GAUT8* and *GAUT12* exhibited dwarf phenotype with disruption of vascular bundle development, and reduction of cell adhesion was observed in *gaut8* (Bouton et al., 2002; Orfila et al., 2005; Pena et al., 2007; Persson et al., 2007). Lower amounts of GalA and xylan, a component of hemicellulose in cell walls, were found in *gaut8* and *gaut12* than wild type. HG;GalAT activities were reduced in the microsome fraction isolated from these mutants, but HG;GalAT activity was not detected in GAUT12 *in vitro*. In addition, *AtGATL5* and *AtGAUT11* single mutants exhibited reduction of seed coat mucilage in which RG-I is contained (Caffall et al., 2009; Kong et al., 2013). In these mutants, amounts of GalA and rhamnose (Rha), monosaccharides composed of RG-I backbone, in extracted mucilage were reduced, showing that GATL5 and GAUT11 participate in both of HG and RG-I synthesis and mucilage synthesis. Since the amount and structure of RG-II were not examined in these studies, it is unknown that these GAUTs contribute to synthesis of RG-II backbone. Furthermore, as there is no direct evidence that these GAUTs possess enzymatic activities to transfer GalA to xylan or RG-I as acceptor polysaccharides, it is still possible that the disorders found in xylan and RG-I were just consequence of the secondary effect of reduced HG synthesis. However, assuming their diversity among the gene family, it is highly possible that some members of GAUTs and GATLs have roles in not only HG synthesis but also in synthesis of RG-I and hemicellulose.

To date, there are no reports indicating that mutations of *GAUT* and *GATL* genes affected RG-II synthesis. However, it is highly possible that some of *GAUT* and *GATL* genes are involved in synthesis of HG, corresponding to backbone of RG-II as a HG;GalAT. In addition, as GalA is also present in side chain A, some of them might play a specific role as a GalA transferase to synthesize side chain A.

### Synthesis of RG-II Side Chains

As mentioned above, RG-II is highly complicated but conserved polysaccharides among plant species, which has HG as a backbone and four side chains (A–D) constituted of 12 different monosaccharides. RG-II is covalently cross-linked by borate diester bonds between the apiosyl residues in side chain A (O’Neill et al., 2004; Atmodjo et al., 2013). Through identification of monosaccharide synthases and transferases for RG-II synthesis (Table 1), it is further demonstrated that normal RG-II structure is key to efficient and stable crosslinking of RG-II by borate.

**TABLE 1:** **Experimentally characterized genes involved in RG-II synthesis**.

**Side chain**	**Sugar**	**Synthesis or transfer**	**AGI code**	**Name**	**Enzyme name**	**Mutant phenotype**	**Rescue by high B**	**Reference**
A	GlcA	Synthesis	At3g29630/At5g 15490	UGD2/UGD3	UDP-Glc dehydrogenase	Dwarfism	–	Reboul et al. (2011)
	L-Gal		(At5g28840)	SIGME	GDP-Man 3,5-epimerase	Dwarfism	+	Voxeur et al. (2011)
A,B	L-Fuc	Synthesis	At3g51160	GMD2 (MUR1)	GDP-Man 4,6-dehydratase	Dwarfism	+	O’Neill et al. (2001)
	Api/Xyl		(At2g27860)	NbAXS1	UDP-D-apiose/UDP-D-xylose synthase	Growth arrest	N.D.	Ahn et al. (2006)
B	Xyl	Transfer	At4g01770	RGXT1	(1,3)-α-D-xylosyltransferase	No phenotype	N.D.	Egelund et al. (2006)
			At4g01750	RGXT2			N.D.	
			At1g56550	RGXT3			N.D.	Egelund et al. (2008)
			At4g01220	RGXT4	(1,3)-α-D-xylosyltransferase	Impairment in pollen tube and root	+	Liu et al. (2011)
C	Kdo	Synthesis	At1g79500	KDSA1	Kdo-8-phosphate synthase	Impairment in	N.D.	Delmas et al. (2008)
			At1g16340	KDSA2		pollen tube in double mutant	N.D.	
			At1g53000	CKS	CMP-Kdo synthetase	Impairment in pollen tube	N.D.	Kobayashi et al.(2011)
		Transfer	At5g03770	KDTA	Kdo transferase		N.D.	Seveno et al. (2010)
			At1g08660	SIA1	Sialyltransferase-like protein	Impairment in pollen tube	N.D.	Deng et al. (2010)
			At3g48820	SIA2	Sialyltransferase-like protein	Impairment in pollen tube	N.D.	Dumont et al. (2014)

N.D., not determined.

Side chain A is composed of seven different monosaccharides, L-fucose (Fuc), Api, glucuronic acid (GlcA), L-galactose (Gal), methyl-xylose (Xyl), GalA and Rha. It has been demonstrated that the normal structure of side chain A is essential for borate crosslinking. In *A. thaliana*, *MURUS1* (*MUR1*) encodes GDP-D-mannose-4,6-dehydratase required for synthesis of L-Fuc, a component monosaccharide found in RG-II. In *mur1* mutant plants, inhibition of leaf expansion and reduced RG-II crosslinking rate were observed (O’Neill et al., 2001) possibly because of truncated side chain A (Pabst et al., 2013). Application of B restored leaf expansion of *mur1*, accompanied with increased proportion of RG-II crosslinking (O’Neill et al., 2001). This suggested that RG-II crosslinking by borate and leaf expansion was positively correlated, and borate crosslinking of RG-II was essential for normal leaf expansion. This study with *mur1* is the first example which shows that normal RG-II structure is essential for efficient RG-II crosslinking by borate and plant growth.

Furthermore, in this decade, several key monosaccharide synthases for RG-II side chains have been identified through genetic studies. Api is present in both side chains A and B and apiosyl residues in side chain A are the sites where borate diester bonds are formed. UDP-Api is synthesized by UDP-D-Api/UDP-D-Xyl synthase (AXS1; Molhoj et al., 2003). In *Nicotiana benthamiana*, silencing of *AXS1* expression caused arrest of plant growth associated with decrease in amounts of side chains A and B (Ahn et al., 2006). UDP-glucuronic acid is a key precursor for production in Ara, Xyl, GlcA, and Api found in cell walls including RG-II. Synthesis of UDP-glucuronic acid is mediate by UDP-glucose dehydrogenase (UGD). The double mutant of *UGD2* and *UGD3* led to dwarfism and impaired fertility with remarkable reduction of side chains A and B (Reboul et al., 2011).

L-Gal is a monosaccharide present in side chain A. Silencing of *GME*, GDP-D-mannose 3,5-epimerase, required for L-Gal synthesis caused dwarfism associated with reduction of L-Gal in cell walls and RG-II crosslinking rate in tomato (Gilbert et al., 2009; Voxeur et al., 2011). Importantly, high level of B supply rescued growth defects of *GME*-silenced tomato plants, accompanied with recovery of RG-II crosslinking rate, similar to the case of *A. thaliana mur1* mutants. In addition, *RGXT1*, *RGXT2*, and *RGXT3* were found to encode (1,3)-α-D-xylosyltransferase capable of transferring Xyl to L-Fuc *in vitro*, probably for synthesis of side chain A (Egelund et al., 2006, 2008). Among the four *RGXT* members, mutations in *RGXT4* were shown to be responsible for *male gametophyte defective 4* (*mgp4*) mutant phenotype in *A. thaliana* (Liu et al., 2011). Mutations in *RGXT4* inhibited elongation of roots and pollen tube, and reduced Xyl amount and RG-II dimerization by 20% in cell wall fraction compared to wild type plants (Liu et al., 2011). Growth disorders of *rgxt4* mutant plants were also restored by high B application to increase proportion of RG-II crosslinking, suggesting that RGXT4 is also involved in synthesis of side chain A. Growth recovery in these mutants by high B supply further supports a view that reduced RG-II crosslinking impairs plant growth and that normal structure of side chain A is important for formation of borate crosslinked RG-II.

RG-II contains an eight carbon sugar, 3-deoxy-D-*manno*-2-octulosonic acid (Kdo) in side chain C, probably unique to RG-II in plants. Kdo is a component of lipid A in Gram-negative bacteria. Recent reports raise a possibility that lipid A-like molecules are synthesized even in plants, however, it is not yet confirmed that Kdo is included. Kdo is synthesized as Kdo-8-phosphate by KDSA encoding Kdo-8-phosphate synthase. After dephosphorylation, Kdo is converted to activated form, CMP-Kdo, by CMP-KDO synthetase (CKS) encoding CTP:KDO cytidylyltransferase. The double mutant of *AtKDSA1* and *AtKDSA2* was not obtained due to impaired pollen tube elongation (Delmas et al., 2008). Similarly, insertional mutations in *CKS* gene inhibited pollen tube elongation, resulting in absence of homozygous mutant plants (Kobayashi et al., 2011). It should be noted that recombinant CKS protein exhibited the enzymatic activity *in vitro*, and is localized in mitochondria but not Golgi lumen *in planta* (Kobayashi et al., 2011). It was found that a putative Kdo transferase KDTA was also localized in mitochondria, but the RG-II structure was not affected in the null-mutant of *KDTA*, unlikely involved in RG-II synthesis (Seveno et al., 2010). Recently, bioinformatics analysis has made a list of candidate genes involved in RG-II synthesis by co-expression analysis (Voxeur et al., 2012). Among them, two putative sialyltransferase-like proteins (SIA1, SIA2) were included and mutations of the corresponding genes were found to inhibit pollen tube elongation (Deng et al., 2010; Dumont et al., 2014). Although there is no experimental evidence for enzymatic activity, it is suggested that these genes, rather than the gene designated as *KDTA*, encode enzymes to transfer Kdo in side chain C and/or 3-deoxy-D-*lyxo*-2-heptulosaric acid (Dha) in side chain D, which has similar characteristics to Kdo.

As described above, enzymes required for RG-II synthesis has been increasingly identified but identification of most of the transferases is still missing in the pathway. Bioinformatic analysis is expected to become a powerful tool to identify novel monosaccharide synthases and transferases that specifically function in RG-II synthesis as mentioned (Voxeur et al., 2012). One interesting feature in some loss-of-function mutants is growth recovery by exogenous application of B associated with increased proportion of RG-II crosslinking possibly due to change of movement of chemical equilibrium (monomeric RG-II + borate ⇄ borate dimerized RG-II). This observation suggests that incomplete structure of RG-II reduces dimerization efficiency or stability of dimerized RG-II, and this highly complicated structure is required for RG-II dimerization and its function. It is possible that mutant plants with altered B nutrient properties might include mutants defective in RG-II synthesis. Further identification of the enzyme and its localization will reveal whole pathway of RG-II synthesis.

## Formation of Borate Crosslinking of RG-II

The timing, the location and the mechanisms that underlie dimerization of two RG-II polysaccharides by borate are still unknown, after RG-II is synthesized in Golgi. Two possible sites for the dimerization reaction are Golgi and apoplast, however, in either case, organization of pectic networks with dimerized RG-II seems puzzling. If RG-II is cross-linked inside Golgi prior to secretion to apoplast, it is unclear how cross-linked RG-II complex is precisely incorporated in two independent HG chains. Alternatively, if borate crosslinking is formed in apoplast, how can two RG-II polysaccharides efficiently find each other to make 90% of RG-II crosslinked in cell wall?

To date, the presence of enzymes catalyzing borate crosslinking of RG-II has not been revealed (Figure 1B). It has been demonstrated that borate crosslinking can occur only with isolated RG-II and boric acid in the presence of divalent cations *in vitro* (O’Neill et al., 1996), implying that any particular molecule/enzyme is not necessarily required for the reaction. However, these reconstitution experiments were conducted with isolated RG-II by endopolygalacturonase digestion and relatively higher B concentration at mM level. It is known that the B concentration in root cell sap of *A. thaliana* grown under normal B conditions is in the range of μM level (Takano et al., 2002). The reconstitution experiments conducted in the previous studies may not reflect physiological situations in plant cells. For the RG-II dimerization to occur under the physiological concentration of B, it is likely that molecules/enzymes are present that facilitate and/or stabilize borate crosslinking of RG-II *in planta*.

One recent study with *Rosa* cultured cells provides a significant implication for sites and timing of this reaction (Chormova et al., 2014). In this report, a novel method to detect dimeric and monomeric RG-II by gel electrophoresis was first established. As HPLC connected with refractive index detector has been widely used, this technique is quite useful to compare a series of samples in one gel. It was uncovered that application of boric acid in media increased dimeric RG-II but preexisting monomeric RG-II remained in *Rosa* cultured cells, suggesting that newly synthesized RG-II is capable to form borate dimerized RG-II. This idea was further supported by the observation that RG-II domain supplied in media did not form dimerized RG-II and was not incorporated to preexisting RG-II in cell walls. These results clearly show that borate crosslinking occurs during RG-II synthesis in Golgi and/or secretion into apoplast.

Another recent report from the same group raises a possibility that glycosylinositol phosphorylceramides (GIPCs) present in lipid raft are able to promote borate RG-II dimerization with the use of *Rosa* cultured cell (Voxeur and Fry, 2014). The experiment by paper electrophoresis pointed out the possible interaction between GIPC and RG-II in the presence of boric acid *in vitro*, implying the formation of complex of GIPC and RG-II linked by borate. More importantly, application of GIPCs was found to enhance borate dimerization of RG-II *in vitro*. Although these experiments were performed *in vitro*, this study provides a new hypothesis for the process of RG-II dimerization. In addition to borate dimerization of RG-II, degradation of borate crosslinking and RG-II turnover will be an intriguing point.

## Boron Transporters for Efficient RG-II Crosslinking

How is boric acid/borate supplied to RG-II to form dimerized RG-II? Boric acid utilized in the cell wall is derived from boric acid present in soil solution. B is mainly present as boric acid in soil solution and plants uptake boric acid from soil into root cells, and translocate into aerial portion of the plants through xylem. Since boric acid is a small non-charged molecule under physiological pH (p*K*_a_ 9.24), it shows relatively high permeability to lipid membrane compared to the other minerals. Based on this, it had been long considered that boric acid was transported only by passive diffusion across membrane along with transpiration stream within plants. Recent studies have identified two types of B transport molecules required for efficient transport of boric acid under limited B conditions, directly affecting borate crosslinking of RG-II.

### NIPs, Boric Acid Channels for Efficient Transport of B

One type is NIPs. NIPs are boric acid channels belonging to major intrinsic protein (MIP) family which includes aquaporins. In *A. thaliana*, NIP5;1 was first identified as a boric acid channel which facilitates uptake of boric acid from external environments into root cells under low B conditions (Takano et al., 2006). NIP5;1 is localized to outer domain of plasma membrane in root epidermis of root tip and elongation zone (Takano et al., 2010). Under limited B conditions, *A. thaliana* mutant plants of *NIP5;1* exhibited lower B concentrations in root and shoot tissues, and inhibition of root cell elongation and leaf expansion. These impaired growth is considered to be caused by reduction of borate cross-linked RG-II as high B supply in media increased B concentrations in these tissues and rescued the growth suppression. It suggests that initial uptake of boric acid is an essential process to satisfy the B demand in the whole tissues to establish borate crosslinked RG-II when B concentrations in media is limited.

*A. thaliana NIP6;1*, a paralogous gene to *NIP5;1*, is shown to participate in preferential distribution of boric acid to growing leaves (Tanaka et al., 2008). NIP6;1 is expressed in vascular bundles particularly in phloem regions including companion cells. Disruption of *NIP6;1* restricts B distribution in young leaves, consequently causing inhibition of upper leaf expansion. Assuming that crosslinking of RG-II by borate is required when new cell walls are constructed in the developing cells, boric acid should be continuously supplied to the sink organs in order to maintain RG-II crosslinking in the newly emerging cells.

Recently, homologs in other plant species, *OsNIP3;1* in *Oryza sativa* and *ZmNIP3;1* in *Zea mays*, have been shown to play similar roles to *AtNIP5;1* and *AtNIP6;1* for efficient uptake and distribution of boric acid under limited B supply. In *OsNIP3;1* RNAi rice plants, the whole plant size was reduced compared to the wild type plants under limited B availability and B distribution patterns in aerial portion of the rice plants was altered (Hanaoka et al., 2014). In maize, *tassel-less1* (*tls1*) was previously isolated as a mutant which exhibited impaired vegetative growth and development of tassels and ears. It was revealed that these growth disorders in *tls1* mutants were caused by mutations in *ZmNIP3;1* encoding a boric acid channel (Durbak et al., 2014; Leonard et al., 2014). In *tsl1* mutants, growth defects including reduced size of shoot apical meristem and impaired development of inflorescence were observed, and reduction of borate dimerized RG-II in immature tassels was experimentally detected. These phenotypes are obviously found in low-B environments whereas high B application rescued. These studies further confirm that overall uptake and subsequent distribution of boric acid is essential for formation of borate cross-linked RG-II and normal growth and development.

### BORs, B Exporters for Efficient Translocation of B

The other type is a boric acid /borate efflux-type transporter BOR1 which shows similarity to anion exchangers found in yeast and animals. *A. thaliana* BOR1 was first identified from *bor1-1* mutant plants defective in xylem loading in roots and preferential distribution of B into young leaves in shoots (Takano et al., 2001, 2002). BOR1 is predominately expressed in inner domain of plasma membrane of endodermis in roots (Takano et al., 2010) and is crucial for efficient translocation of B from roots to shoots to maintain B levels in leaves under B starvation. In *bor1-1* mutant plants, the B concentrations in shoots are dramatically decreased and expansion of upper leaves is severely inhibited under limited B availability. In *bor1-1*, it is revealed that sugar composition of RG-II is not affected and proportion of borate dimerized RG-II is reduced to 40% whereas 90% of RG-II is crosslinked in wild type plants (Noguchi et al., 2003). These results support an idea that RG-II synthesis and B nutrient status do not affect each other, and further the local B concentration in the cells is one determinant for crosslinking of RG-II.

*BOR2*, the most similar to *BOR1* in *A. thaliana* genome, is considered to be responsible for cellular distribution of B to RG-II in roots. Loss of function of *BOR2* inhibits root cell elongation and decreases relative proportion of RG-II crosslinking in roots although the total B concentrations in roots were not significantly different from those of wild type plants under limited B conditions (Miwa et al., 2013). This finding suggests that BOR2 is involved in preferential distribution of B into RG-II at the cellular level under low B conditions. BOR2 appears to be predominately localized in plasma membrane and be recycled between plasma membrane and endosomes for polar localization in inner domain of plasma membrane. Although molecular mechanisms underlying B transport into RG-II through BOR2 are not yet determined, localization of BOR2 raises a possibility that incorporation of B into RG-II could be induced either in plasma membrane or in endosome/secretion vesicles.

As one possible hypothesis, when we assume that BOR2 localized in plasma membrane exports B into RG-II in apoplast, borate crosslinking of RG-II could occur beneath the plasma membrane in apoplast. In this case, direct or indirect interactions between BOR2 and RG-II monomer or between BOR2 and facilitators including GIPCs might be necessary for efficient delivery of B to RG-II monomer because exported boric acid/borate by BOR2 into apoplast could easily return back to cytosol due to the high permeability. Interaction between BOR2 and GIPCs present in plasma membrane for efficient RG-II dimerization would be an interesting hypothesis.

As the second possible hypothesis, BOR2 localized in endosomes/secretion vesicles could increase B concentrations in these vesicles to enhance the crosslinking reaction inside the vesicles, since BOR2 in the vesicles is likely able to transport B from cytosol into the vesicles. Among the BOR family in *A. thaliana*, BOR4, encoding a B exporter, is also localized to plasma membrane in roots but functions in B exclusion out of the cells against B toxicity when B is present in excess (Miwa et al., 2007, 2014). Overexpression of BOR4 confers high-B tolerance but results in higher sensitivity to B deficiency due to lower B concentrations in roots. Assuming that BOR4 may not be localized to endosomes, the finding possibly implies that localization of B exporters in endosomes/secretion vesicles is required for B delivery to RG-II by increasing B concentrations inside the vesicles. This second hypothesis supports a view suggested by Chormova et al. (2014) that borate crosslinking of RG-II occurs during RG-II synthesis in Golgi or secretion.

Orthologous genes to *AtBOR1* have been increasingly identified in *O. sativa* (Nakagawa et al., 2007), in *Triticum aestivum* (Leaungthitikanchana et al., 2013), *Vitis vinifera* (Perez-Castro et al., 2012), and *Z. mays* (Chatterjee et al., 2014), suggesting that *BOR1* genes are required for efficient B translocation to ensure sufficient supply for RG-II dimerization under B starvation in a wide range of plant species. In maize mutant named *rotten ear*, impaired development of tassels and ears was proven to be attributed to the point mutations in *ZmBOR1/RTE* (Chatterjee et al., 2014). Observation by TEM clarified that organization of cell walls was disrupted and cell adhesion was loosened in the ears of the *rte* mutant. Exogenous application of high B restored these adverse effects. Together with the study of *tls1* maize mutants, these researches support that optimization of B transport by B transporters is required for normal development of inflorescence and fertility even in monocot species, which generally require lower amount of B compared to dicots because of lower amount of pectin in cell walls. In addition, *OsBOR4*, one of four *BOR* genes present in *O. sativa*, is found to be specifically expressed in pollen (Tanaka et al., 2013). Rice plants homozygous for Tos17 transposon insertion were obtained and they exhibited fertility comparable to wild type plants, however, occurrence of the mutant homozygous plants was lower than expected in the progeny derived from self-fertilization of the heterozygous mutant plants and germination rate of mutant pollens in pistils were lower than those of wild type plants. Assuming that high amount of B needs to accumulate in the growing tip of pollen tube (Iwai et al., 2006), B transporters locally deliver boric acid possibly into RG-II in pollen, resulting in regulation of germination and/or elongation of pollen, and consequent fertilization.

## Perspectives

We still have a number of questions to address: How are the four side chains of RG-II synthesized and the structure is organized? Is there any enzyme that facilitates borate crosslinking? How does “borate crosslinked RG-II” regulate cell elongation? Does borate cross-linked RG-II not only determine cell wall properties but also function as a signaling component to regulate growth?

For gelation of pectin, Ca^2+^ bridges between HG polysaccharides might be sufficient. Why have plants evolved to utilize B, such a unique element, and RG-II for cell walls? Plants have developed defense systems against pathogens including fungi that are able to degrade HG by the activities of pectin methylesterase and endopolygalacturonase. Plant cell wall is the frontiers in the competition between plants and pathogens, and therefore plants have mechanisms in which oligo-galacturonide, degraded HG, functions as a signal molecule that represents disruption of cell walls. Since borate crosslinking has not been reported to be hydrolyzed by fungi, borate crosslinked RG-II could be free from the attack by fungi and prevent complete degradation of pectic polysaccharides. Evolutionary aspects in occurrence of cell wall degradation enzymes in fungi and novel components in plant cell wall should be focused.

Further identification of novel players for borate crosslinking of RG-II, such as GT, a membrane lipid and B transporters, will lead to understanding a whole figure of synthesis and physiological impacts of borate crosslinked RG-II in plants.

### Conflict of Interest Statement

The authors declare that the research was conducted in the absence of any commercial or financial relationships that could be construed as a potential conflict of interest.
